# Organic Mulch Increases Insect Herbivory by the Flea Beetle Species, *Disonycha glabrata*, on *Amaranthus* spp.

**DOI:** 10.3390/insects11030162

**Published:** 2020-03-03

**Authors:** Roger V. Vorsah, Beatrice N. Dingha, Sudan Gyawaly, Sarah A. Fremah, Harmandeep Sharma, Arnab Bhowmik, Mulumebet Worku, Louis E. Jackai

**Affiliations:** 1 Department of Natural Resources and Environmental Design, North Carolina Agricultural & Technical State University, Greensboro, NC 27411, USA; rvvorsah@aggies.ncat.edu (R.V.V.); bndingha@ncat.edu (B.N.D.); gyawaly17@gmail.com (S.G.); hsharma@ncat.edu (H.S.); abhowmik@ncat.edu (A.B.); 2 Department of Animal Science, North Carolina Agricultural & Technical State University, Greensboro, NC 27411, USA; sadjeifr@ncat.edu (S.A.F.); worku@ncat.edu (M.W.)

**Keywords:** Amaranth, flea beetle, *Disonycha glabrata*, organic, integrated pest management, mulch

## Abstract

Amaranth (*Amaranthus* spp.) is an increasingly high-valued niche vegetable crop among small organic growers in North Carolina, due to its increasing demand among diverse immigrant groups. Production is however hampered by insect pests such as the flea beetle (FB), *Disonycha* *glabrata* (Coleoptera: Chrysomelidae), that cause significant yield reduction. Chemical insecticides are generally applied for pest control despite their known risks to health and the environment. Integrated pest management (IPM), which is a cost effective and environmentally friendly approach is still under-exploited in vegetable production by small growers. We studied IPM approaches, suitable for organic production of amaranth by screening nine amaranth varieties for resistance to the flea beetle (FB), *D*. *glabrata*, grown with, and without, mulch. *D. glabrata* population was 60% higher in plots with mulch compared to plots without. The amaranth varieties *Molten fire* and *Green Callaloo* recorded the lowest and the highest *beetle* population commensurate with low, and high leaf damage, respectively. Conversely, leaf yields in the mulched plots were 50% less than recorded in the zero-mulch counterpart, with *Green Callaloo* variety recording the lowest. These findings will serve as building blocks for a sustainable pest management plan that is appropriate for organic production of *Amaranthus* spp. in North Carolina.

## 1. Introduction

Global efforts to feed the ever-growing world population that is predicted to reach 8.1 billion by 2025 [1], and the need to attain food security have galvanized the drive to cultivate crops on various scales, including small backyard and community gardens that are mostly organic culture, both in rural areas and among urban dwellers (the new small farmer community) [2,3,4]. A multiple of commodities are grown in these farms, 67.5% of them fresh vegetables including amaranth greens that are the subject of this paper [2,3]. Organic vegetable farming faces many challenges [5,6,7]. Insect pests, weeds and diseases are among the major constraints in these systems, and some of the practices that have been adopted to solve them tend to be production system-specific. Organic production practices use methods of pest control that limit, or completely eliminate, the use of high-risk synthetic pesticides and benefit from the use of IPM approaches including the use of various mulches and other biological and cultural approaches, *inter alia* [8,9]. These approaches seek to mitigate the impact of pests, delay the development of insecticide resistance, curb environmental contamination, and minimize human health risks [10]. Some successes have been reported for example by MacHardy [8] and Nottingham et al. [9] in fruit orchards and vegetables. However, organic mulches have not been evaluated in the production of amaranth in organic systems in N. Carolina despite their popularity among organic growers, especially those in urban areas.

Different types of mulches have been used in agriculture for decades and they have become commonplace in the cultivation of a wide range of crops worldwide, gaining prominence especially in organic systems as a sustainable management practice for weeds and pests, as well as other ecological services [11,12,13]. Mulches are essentially an environmental diversification tactic that alters the ground habitats of arthropods and other organisms, and in turn, impacting crop yields and related variables; in structure, they are as variable in type and content and include organic, non-organic, live plants, synthetic, dead plant-based, biodegradable and non-biodegradable mulches [11,12,14,15]. An important characteristic of mulches that has direct relevance to the present study is their impact on the dynamics of ground-dwelling arthropods and the fact that responses to their use is both species- and mulch type-dependent. Reports on the use of mulches in agricultural crops appear to show two broad trends when compared with bare-ground controls: (i) Pest populations and crop damage are reduced (in part as a result of increased natural enemy activity) [13,16,17]; (ii) pests and their associated damage are increased (probably from enhanced micro-environment for the pest, interference with natural enemy activity and other reasons) [7,13,18]. Since yields tend to increase in the former unlike in the latter, the decision to include mulch in a pest management system can become quite important, particularly when combined with other IPM tactics, such as intercropping, genetic resistance and row covers [19].

Mulches are widely used by small growers in North Carolina (NC) where transitioning to sustainable or organic production has increased (72 farms in 2012 to 87 farms in 2017). This shows a clear tipping of the scales towards consumer demand for organic products [20]. Small growers in NC grow multiple crops, mostly vegetables and fruits, on limited (often leased) farmland averaging 2 to 10 acres [20]. Specialty vegetables like *Amaranthus* spp. and quinoa have gained rapid acceptance among small growers in the state as demand for them increases, especially among immigrants and some health-conscious individuals [21]. Amaranth is among the three most important “pseudocereals” of worldwide importance [22]. *Amaranthus* spp. (commonly known as pigweed in the US) are annuals or short-lived perennials with over sixty species, 4–6 thousand varieties but only a few species are cultivated for food (e.g., *Amaranthus hypochondriacus*, a. *caudatus*, a. *cruentus*, a. *retroflexus*) [21]. *Amaranthus spp*. are cultivated either for the leaves, very young and tender whole plants or for grains. The plant contains high levels of minerals, vitamins, proteins, antioxidants and are gluten-free making it a perfect choice in overcoming many health-related disorders in humans [23]. Several insect pests in the following orders attack and cause economic losses on *Amaranthus* spp.; the most prominent are Coleoptera, Lepidoptera, Hemiptera and Diptera [23,24]. In North Carolina and elsewhere in the USA the flea beetle, *Disonycha* spp. (Coleoptera: Chrysomelidae) is the most important. *Disonycha* spp. generally feeds on the lower leaf surface avoiding the veins and creating a skeletonized appearance of the leaf [25,26,27]. Young plants and seedlings are particularly susceptible, resulting in growth retardation and subsequent death, due to a drastic decrease in chlorophyll content and desiccation [28]. This subsequently leads to loss of yield and income as no one buys heavily damaged leaves. In order to mitigate losses, organic growers in other countries have tried several management tactics including the use of mulch and resistant varieties for their lepidopteran defoliators (e.g., *Spoladea recurvalis* Fab., Family Crambidae) with varying success [29,30,31]. In North Carolina, Lepidoptera are not a major problem on amaranth. In addition, the varieties and mulch treatment (leaf compost mulch) have not been evaluated anywhere else on this crop. Consequently, we could not determine whether previous findings would apply to flea beetles which, unlike in the lepidoptera, cause defoliation both as adult and to a much less extent by larva. The present study was therefore developed to investigate the existence of amaranth resistance to *D. glabrata* feeding and the underlying effects of compost mulch on the overall productivity of amaranth. As indicated earlier, mulch is known to have variable effects on different insects under different conditions [6,13]. Leaf compost mulch (which is available free-of-charge) is favored among organic growers in Greensboro, N. Carolina where this study was conducted. Considering that this insect breeds in the soil and emerges to cause defoliation mainly as adults, it was important to determine the plant and/or soil phytobiome interactions at the plant-insect-environment interface that will result in positive outcome from the combined use of resistant varieties and compost leaf mulch.

## 2. Materials and Methods

### 2.1. Field Screening for Resistance

The study was conducted at the Guildford College Organic Farm (GC-Farm) in Greensboro, NC (longitude 36.0987° N and latitude 79.8884° W). The experiment was set up in a split-plot experimental design with the main plot factors being (i) leaf mulch and (ii) without leaf mulch, and subplot factors being the (9) amaranth varieties [*Tricolor* (TR), *Green Callaloo* (GC), *Hopi Red Dye* (HR-D), *Mayo Indian* (MI), *Red Garnet* (RG), *Molten Fire* (MF), *Opopeo* (OP), *Red Leaf* (RL), and *Golden Giant* (GG)]. A 2-m buffer separated main plots each measuring 15 m x 11m while subplots measuring 4 m x 1.5 m were 1.5 m apart. The experiment had three replications. Table 1 shows the morphological descriptions of the amaranth varieties used in the study.

Amaranth varieties were selected based on their adaptation to NC conditions according to the 2017 Southeastern Vegetable Handbook [32]. Seeds were purchased from Johnny’s Selected Seeds (Winslow, ME), Stoke Seeds Ltd. (Thorold, ON, Canada) and Baker Creek Heirloom Seed (Mansfield, MO, USA). The leaf mulch was obtained from White Street Landfill in Greensboro, NC. A custom-formulated soil mixture (any other planting soil would also work) was used for sowing the seeds in the greenhouse at Guilford College Farm in Greensboro, under natural light conditions on 2 May in seventy-two-cell seed trays (54 × 29 × 6 cm), three seeds per cell. Temperature was maintained between 18 °C and 24 °C and relative humidity between 70% and 80%. Germination was observed six to eight days after sowing (DAS). At 10 DAS, we transferred one plant per cell into thirty-six-cell seed trays (27 × 15 × 6 cm) to avoid overcrowding as well as ensure optimum plant growth. Plants were transplanted in the field on 5, and 6 June in 2017, and 2018, respectively. During transplanting, 3 mL cup of organic material research institute (OMRI) certified all-natural composted poultry layer manure, Harmony^®^ Ag Organic fertilizer (N-P-K, 5-4-3 with 9% Ca) (Environmental Products LLC, Roanoke, VA) was added to the soil. Planting density was 8 plants per 4 m row at a within-row plant distance of 0.5 m. All weeding was done manually. In 2017, *D. glabrata* infestation was extremely high and accompanied by early and severe plant damage, making it necessary to apply a preventive spray of PyGanic^®^, an OMRI-approved insecticide with high efficacy against flea beetles. We applied the insecticide once at 50 days after planting (DAP) to prevent total loss of the experiment. The study further assessed optimal presumptive schedules for interventions with insecticide application using a pre-set action threshold (a leaf damage score of 2), for plots with, and without, mulch to avert major yield loss.

### 2.2. Insect Sampling

Insects were sampled 45 DAP on both main and subplots by assessing the above-ground plant parts in each row for presence of the pest. Sampling was focused mainly on pests of amaranth. However, other captured insects were also noted. Insect sampling was conducted by direct visual sampling, double-sided sticky traps and vacuum sampling using a modified leaf blower-vac suction [33]. Direct visual sampling occurred on a pre-determined 2 m row-length in the center of each row from 19 to 26 June; sticky trap sampling was carried out by placing two-sided yellow sticky traps (8 × 13 cm^2^) (Pestrap^™^, Phytotronics Inc., Earth City, MO, USA) affixed on two separate metal stakes set 2 m apart on the row. This was aimed at capturing any smaller insects that we may have missed with visual sampling. We adjusted trap height to keep them just below canopy height. Traps were removed after 24 h and replaced weekly from 3 to 24 July. The highly mobile feeding behavior of *Disonycha* spp. warranted the use of the reversed leaf blower to get a more reliable estimate of their population. The reversed leaf blower (model ECHO ES-250) was fitted with an HDX 5-gallon elastic top strainer (The Home Depot^®^) and then used to sample insects for 30 s per row, between 9 am to 12 pm, from 31 July to 21 August. All samples were transferred into labelled Ziploc^®^ bags and taken to the IPM laboratory where they were identified using a microscope (AmScope Stereozoom trinocular microscope, SZMT2 Series, WF10X/20; United Scope LLC, Irvine, CA).

### 2.3. Leaf Damage Measurement

Leaf damage due to beetle feeding was estimated visually in the field using a damage score of 1–5 (where 1 = 0–20%, 2 ≧ 20–40 %, 3 ≧ 40–60 %, 4 ≧ 60–80 % and 5 ≧ 80–100% leaf damage) modified from the procedure described by Niveyro et al. [24] as follows: Five fully expanded leaves at the crown of each of eight plants on the row were used for damage assessment. To avoid sampling previously sampled leaves, a notch was made on the margins of sampled leaves. This ensured that we sampled only newly opened leaves on subsequent weekly sampling dates.

### 2.4. Determination of Soil Moisture Content in Plots with and without Leaf Compost Mulch

Variation in soil moisture, due to mulching, has been reported to influence the population of insects that spend part, or all, of their life cycle in the soil by impacting egg viability, eclosion, larval and pupal development [28]. Soil moisture analysis was carried out to determine if there were any differences between the two mulch regimens, and how that affected *D. glabrata* populations and damage to amaranth. Soil sample cores (0–30 cm depth) were taken from plots with and without leaf compost mulch at 50 DAP during the summer of 2018. Two soil samples were collected per row per main factor treatment. The initial soil weight of each sample was recorded using a scale (MS6002S Mettler Toledo, Columbus, OH, USA) and then dried in an oven (Isotemp 725G, Fisher Scientific, Waltham, MA, USA) for 48 h at 105 °C. After drying, the soils were weighed again. Soil gravimetric moisture (GMC) content was determined as follows,

(1) $$GMC=\frac{M_{1}-M_{2}}{M_{2}-\;M_{3}}\;\times \;100$$

where: *GMC* = Gravimetric Moisture Content, *M*_1_ = Weight of moist soil plus can, *M*_2_ = Weight of dry soil plus can, and *M*_3_ = Weight of empty can

### 2.5. Determination of Total Leaf Protein and Polyphenol Concentration among the Amaranthus spp.

Plant proteins, polyphenols and other secondary metabolites constitute a suite of defense compounds in plants. Some work has been reported on these compounds in *Amaranthus* spp. and lepidopteran pests [24,31]. In our study, we investigated the effects of total leaf protein and polyphenol concentrations in amaranth varieties to enable us explain observed differences in *D. glabrata* herbivory.

#### 2.5.1. Sample Preparation

Fresh amaranth leaf samples were harvested 40 DAP from the nine varieties and stored separately in 50 mL polypropylene centrifuge tubes at −80 °C before analysis.

#### 2.5.2. Determination of Total Leaf Protein Concentration in the Amaranthus Varieties

We weighed a three grams portion of the leaves of each amaranth variety using a digital scale into a 1.5 mL Eppendorf safe-lock micro-centrifuge tube. One (1) mL phosphate buffer saline (PBS) solution was added to the samples and homogenized. The homogenate was centrifuged at 10,000 rpm for 10 min. The pellet was discarded, and the supernatant extracted and transferred into newly labeled centrifuge tubes that were used to determine total protein concentration in the leaf. The Pierce Bicinchoninic Acid (BCA) Protein Assay Kit (Thermo-Scientific, Waltham, MA) was used to determine the total protein concentration of the samples following the manufacturer’s protocol as described previously [34,35,36,37]. Bovine serum Albumin (BSA) of known concentration prepared from an albumin standard was used to help quantify the leaf protein concentration. A 10× and 100× dilution of the samples were prepared along with the albumin standard dilutions. A dilution of 25 µL of the albumin standard dilutions and sample replicates were pipetted into the wells of a 96 well microplate. A working reagent (prepared from two reagents contained in the protein assay kit: Reagent A containing sodium carbonate, sodium bicarbonate, bicinchoninic acid and sodium tartrate in 0.1 M sodium hydroxide and Reagent B containing 4% cupric sulfate) was added to each well (200 µL) and mixed thoroughly on a plate shaker. The plates were covered and incubated at 37 °C for 30 min. Plates were cooled to room temperature and the absorbance was measured at 562 nm using a plate reading spectrophotometer (BioTek™ Epoch™ Microplate Spectrophotometer). A standard curve was plotted from the readings of the albumin standards to determine their protein concentrations.

#### 2.5.3. Determination of Total Leaf Polyphenol Concentration in Amaranthus Varieties

Three grams of frozen amaranth leaves were macerated to powder form in liquid nitrogen using a mortar and pestle. Powdered samples were transferred into 15 mL polypropylene centrifuge tubes. 10 mL of 80% methanol (*w*/*w*) was added to the samples in the tubes. The mixture was incubated at 37 °C for 2 h while shaking in an incubator shaker. The samples were centrifuged at 4000 rpm for 15 min after incubation and the supernatant collected by sieving it through Whatman paper no. 4. The total phenolic content of the methanol extracts was determined using the Folin-Ciocalteu method. Absorbance was measured at 765 nm using plate reading spectrophotometer. Gallic acid was used as a standard. The total phenolic compound was expressed as mg Gallic Acid Equivalent (GAE) per gram.

### 2.6. Amaranth Leaf and Grain Yield

Fresh amaranth leaves together with the subtending bouquet stems were harvested weekly using a hand-held pruner (2.4″ × 1.3″ × 8.5″ Model: F2; The Home Depot^®,^ USA) from 19 July (43 DAP) to 21 August (76 DAP) in both 2017 and 2018. Similarly, partially dried grains on the stems of the grain varieties were also harvested from 70 DAP to 100 DAP during both years using the pruner. Harvested seed heads were air-dried indoors for 3 weeks. The leaves and the dried, threshed grains were weighed using an Ohaus T51P scale (Pine Brook, NJ, USA).

### 2.7. Data Analysis

Diversity in insect species between the main plot factor treatments was determined using the Shannon-Weaver diversity index *(H′*) [38,39],

(2) $$H^{\prime }=-\sum p_{i}\mathrm{In}p_{i}$$

where *H′* = Shannon-Weaver diversity index, p_i_ = the proportion of individuals found in species *i*. p_i_ estimated as,

(3) $$p_{i}=n_{i}/N$$

where *n_i_* = number of individuals in species *i*, *N* = total number of individuals in the community.

A split-plot experimental design was used in this study and repeated measures analysis of variance (ANOVA) was conducted to compare the effects of mulch, variety and year on *D. glabrata* populations. The leaf damage data was analyzed using generalized linear model with poisson distribution and log link function. This was used to compare the effects of mulch and variety on harvestable yield of greens and dry grain yield. The assumptions of normality and homogeneity of variance were analyzed using Shapiro-Wilk, and Levene’s tests, respectively, and data were transformed as needed before analysis. Data on *D. glabrata* populations and feeding damage were log-transformed before the analysis. Significant differences in means were separated using Fisher’s least significant difference (LSD) at 0.05 level of significance. The randomized complete block (RCB) analysis of variance (ANOVA) was used to examine differences in total leaf protein and polyphenol concentration among the amaranth varieties and means were separated using Fisher’s least significant difference (LSD) at 0.05. Pearson’s correlation analysis at 0.05 level of significance enabled us to determine the relationship between: (i) the gravimetric moisture content and *D. glabrata* population; (ii) total leaf protein and *D. glabrata* population; and (iii) total polyphenol concentration and *D. glabrata* population. The assumptions of linearity and homoscedasticity were confirmed using scatter plot, and White’s test, respectively, before correlation analysis. The effect of moisture content, total leaf protein and polyphenol concentration on *D. glabrata* population was analyzed using least square regression at 0.05 significance level. All analysis listed above were conducted using SAS version 9.4 [40].

## 3. Results

### 3.1. Diversity and Abundance of Insects Associated with Amaranth Varieties

In all, 9077 insect specimens were sampled from amaranth plots during the period of June to September 2017 and June to August 2018. The insect species were fell into 6 orders, 22 families and 31 species (Table 2). Hemiptera and Coleoptera had by far the highest abundance of species (43.1% and 43.1%, respectively), followed by Diptera (11.5%), Hymenoptera (1.5%); the least was recorded among the Lepidoptera (0.4%) and Orthoptera (0.4%). The key insect pest that caused the greatest leaf damage on amaranth was the adult flea beetle, *Disonycha glabrata* (Coleoptera: Chrysomelidae). Numerous small round holes that characterized feeding damage by *D. glabrata* were observed on the leaves. The insect samples had low counts of three orders (Coleoptera, Diptera and Hymenoptera) with four families - Coccinelidae, Carabidae, Dolichopodidae and Vespidae that are known predators of different insects. However, none have been reported specifically to be predators of *D. glabrata*.

There was no significant difference (F = 7.34, df = 1, 2; *p* = 0.1135) in insect abundance between the mulch and no mulch treatments in the summer of 2017 despite mulch treatments having 51.3% more insects (Mulch = 3038.0 ± 42.7 per 2m row; no mulch = 1796.0 ± 22.3 per 2m row), neither was there a difference recorded between these treatments in 2018 (F = 9.82, df = 1, 2; *p* = 0.0885) (mulch = 2594.0 ± 35.5 per 2m row; no mulch = 1649.0 ± 19.7 per 2m row), despite a 44.5% more beetles in the mulch treatment. An analysis of species diversity between main plot factor treatments revealed that there was more species diversity in the mulch compared to the no mulch treatments in 2017. Similarly, the species evenness (*E*) in 2017 and 2018 were higher in the mulch treatments and with better species distribution in 2018 (Table 2).

**Figure 1:**
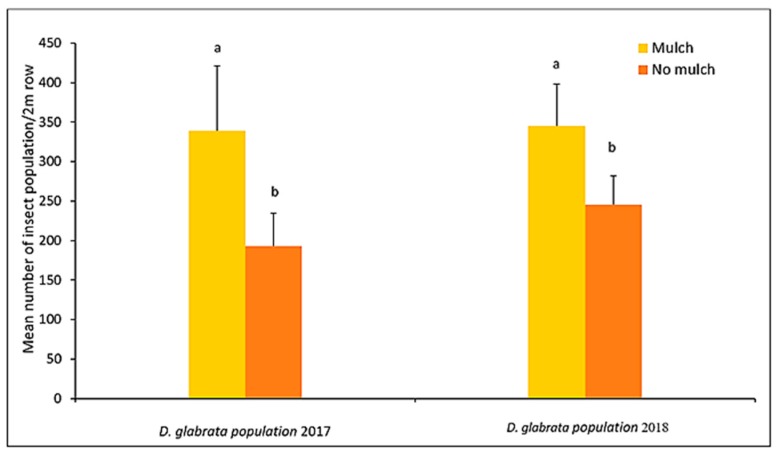
*D. glabrata population* (Mean ± SE) in mulched and without mulched plots during the two-year period. Different letters within the same year indicate significant difference (Fisher’s LSD, *p* < 0.05).

*D. glabrata* was significantly more abundant in the mulch treatments in 2017 (F = 84.42, df = 1, 32; *p* = 0.0191) and in 2018 (F = 232.41, df = 1, 32; *p* < 0.0001) (Figure 1).

**Figure 2:**
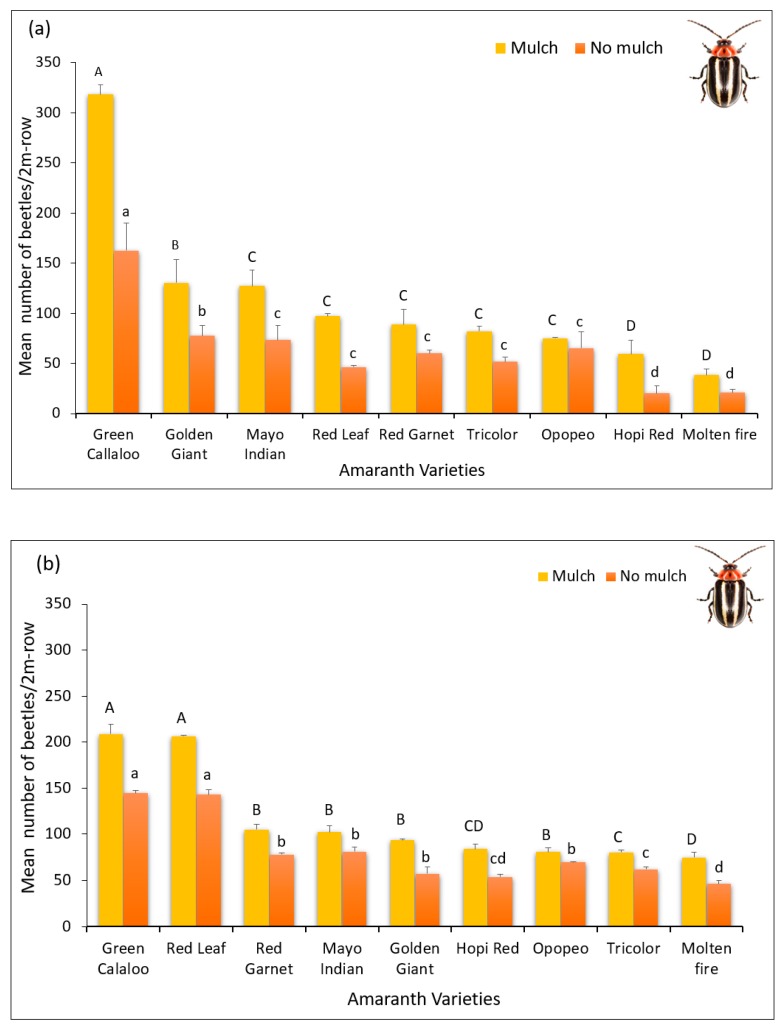
*D. glabrata* population (Mean ± SE) in mulched and no mulched plots in 2017 (**a**) and 2018 (**b**). Different uppercase letters on bars indicate significant difference among varieties within mulch plot treatment, while different lowercase letters above bars indicate significant difference among varieties within no mulch plot treatment (Fisher’s LSD; *p* < 0.05).

There was a highly significant interaction between mulch and the amaranth varieties in 2017 (F = 7.24, df = 8, 32; *p* < 0.0001) as well as in 2018 (F = 7.44, df = 8, 32; *p* < 0.0001). Significant difference was also observed in *D. glabrata* population among the amaranth varieties during both years, in 2017 (F = 60.34, df = 8, 32; *p* < 0.0001) and in 2018 (F = 180.02, df = 8, 32; *p* < 0.0001) with *Green Callaloo* and *Molten Fire*, respectively, recording the highest and lowest beetle population in 2017 and in 2018, under mulch, and without mulch (Figure 2a,b).

### 3.2. Feeding Damage by Disonycha Glabrata

**Figure 3:**
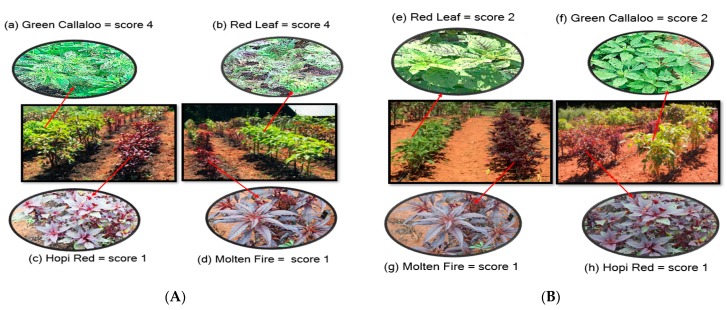
Leaf damage on amaranth varieties on plots with mulch (**A**) (high damage, a and b; low damage, c and d); and on plots with no-mulch (**B**) (high damage, e and f; low damage, g and h).

**Figure 4:**
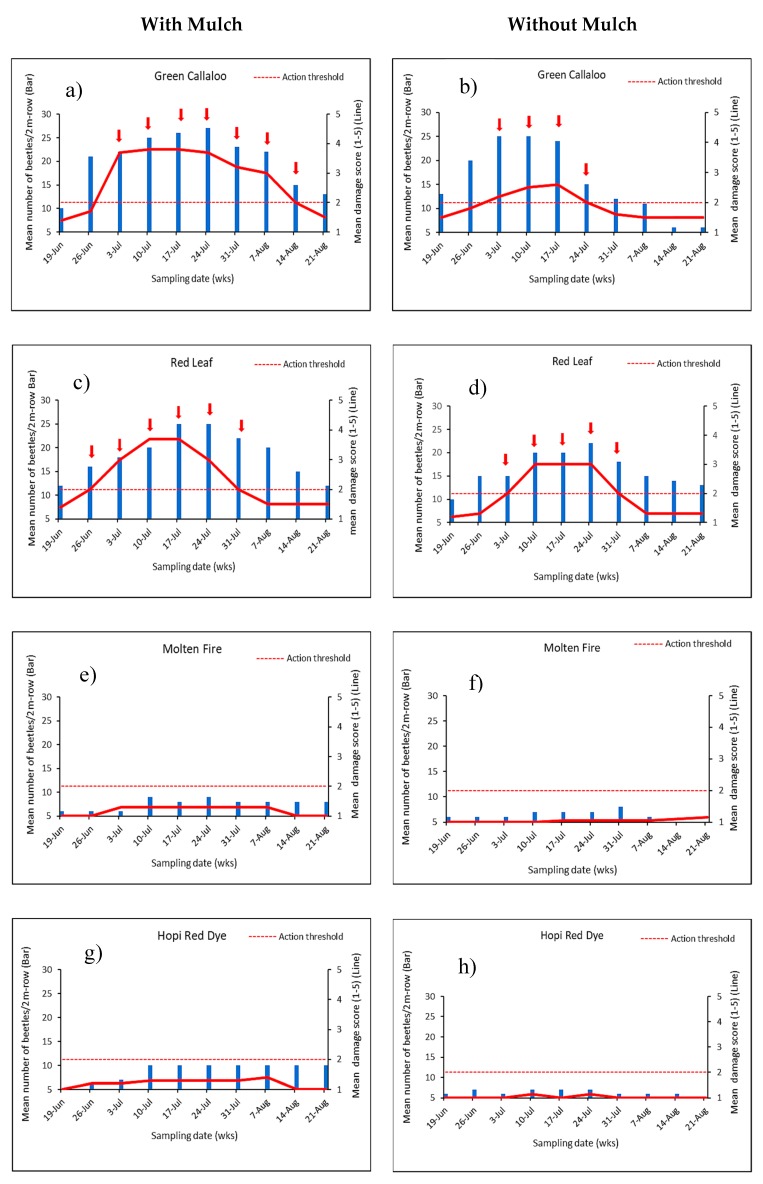
Weekly mean beetle population and the corresponding damage (i.e., scores = 1: 0–20%, 2: >20–40 %, 3: >40–60 %, 4: >60–80 % and 5: >80–100% leaf damage) and the action threshold for each amaranth variety in 2017. Arrows [in a–d] represent presumptive insecticide treatments (≥action threshold score of 2).

**Figure 5:**
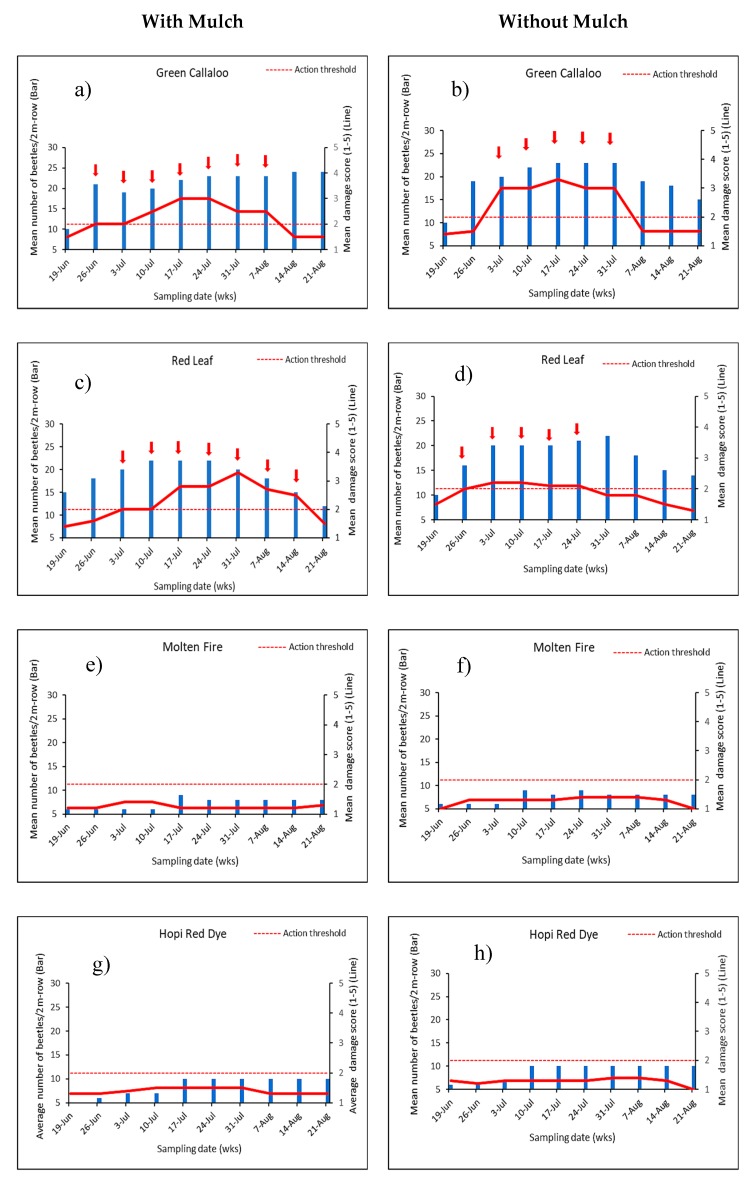
Graphs showing the weekly mean beetle population and the corresponding damage (i.e., scores = 1: 0–20%, 2: >20–40 %, 3: >40–60 %, 4: >60–80 % and 5: >80–100% leaf damage) and the action threshold for each amaranth variety in 2018. Arrows [in a—d] represent presumptive insecticide treatment times (≥action threshold score of 2).

Percentage leaf damage by *D. glabrata* (adults and larvae) varied between 20 to 80% (score of 2–4) among the amaranth varieties (Table 3). The difference in plant damage between mulch and no mulch was significant in 2017 (F = 18.75, df = 1, 32; *p* < 0.0001) with mulch treatment recording higher plant damage (score of 3–4) compared to the treatment without mulch (score of 1–2). Severe and unusually early damage was observed in the entire experiment in 2017 thus warranting a single treatment of PyGanic^®^ insecticide 50 DAP to rescue the study. This was not needed in 2018. Overall plant damage was significantly greater and significant in mulch treated plots (F = 22.62, df = 1, 32; *p* < 0.0001) compared to no mulch plot (Table 3 and Figure 3A,B). The degree of damage was also significantly different among all the varieties in 2017 (F = 10.46, df = 8, 32; *p* < 0.0001) and in 2018 (F = 21.78, df = 8, 32; *p* < 0.0001). The highest plant damage was observed in *Green Callaloo* and *Red Leaf* varieties (Score of 3.0 ≧ 40–60% leaf damage) during both years under mulch and without mulch (Figure 3A,B). On the other hand, *Molten Fire* and *Hop Red Dye* had low scores, 1 (0–20 % leaf damage), during both years under both conditions. Projections of the optimal time for insecticide intervention (presumptive applications) on the amaranth greens with and without mulch to avert major yield loss are presented in Figure 4 and Figure 5. *D. glabrata* population and damage scores obtained in 2017 and 2018 from two susceptible (*Green Callaloo and Red Leaf*) and two resistant (*Molten Fire and Hopi Red Dye*) varieties were used for the analysis.

Figure 4 and Figure 5 show the preset action threshold (score of 2–5 signifying >20–100% leaf damage) that was used to determine the likely need for insecticide treatment, in order to reduce damage associated with *D. glabrata* and achieve greater yield. Recognizing that damage would be much easier for growers than keeping track of insect counts, from the analysis, both *Molten Fire* and *Hopi Red Dye* varieties in mulch and no mulch plots would have potentially required no insecticide application and could be harvested starting from 20 DAP. *Green Callaloo* and *Red Leaf* on the other hand would have required several insecticide applications with PyGanic^®^ based on the working treatment action threshold, a damage score of 2.

### 3.3. Factors Influencing Herbivory between Main PlotTreatments

**Figure 6:**
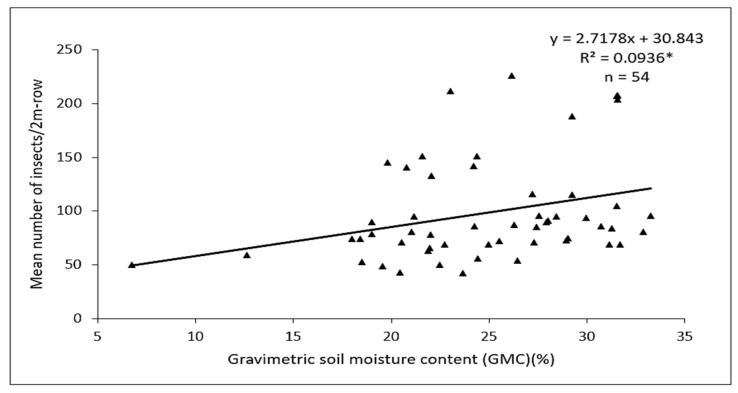
Regression line and equation showing the relationship between gravimetric soil moisture content (GMC) and *D. glabrata* population in 2018. * indicates significant regression coefficient at *p* < 0.05.

Gravimetric soil moisture content (GMC) measured between plots with and without mulch showed significant differences (F = 14.08, df = 1, 32; *p* = 0.0007) between the mulch treatments. Mulched plots recorded a 36% GMC value, which was higher compared to plots without mulch. Pearson correlation coefficient analyses of the 2018 data indicated a positive relationship between *D. glabrata* population and gravimetric soil moisture content (*r* = 0.31; n = 54, *p* = 0.024). Regression analysis of the same data gave an r^2^ value that was significant (*p* < 0.05) with soil moisture content accounting for 9% of the observed variation in *D. glabrata* population (Figure 6).

The regression slope shows that a 1% increase in the soil moisture would cause the D. glabrata population to increase by an average of 2.7 individuals; this would result in an increase in the amount of feeding damage.

### 3.4. Quantification of Total Leaf Protein Content among Amaranth Varieties

**Figure 7:**
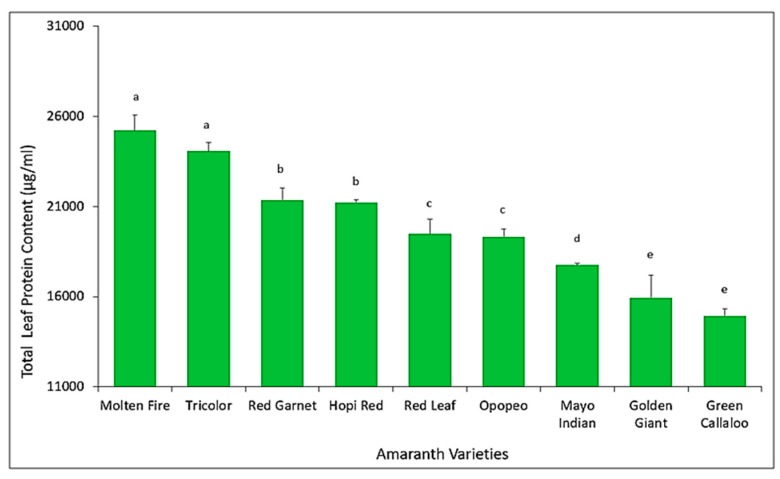
Total leaf protein content (Mean ± SE) among amaranth varieties. Bars with the same letters are not significantly different using Fisher’s LSD (*p* < 0.05).

Results from Pierce BCA Assay derived from the Bovine Serum Albumin (BSA) standard curve show a highly significant (F = 77.76, df = 8, 16; *p* < 0.0001) difference in the total leaf protein concentration among the amaranth varieties. *Molten Fire* recorded the highest total leaf protein concentration (> 21,000 µg/mL) compared to *Green Callaloo* which had the lowest (Figure 7).

**Figure 8:**
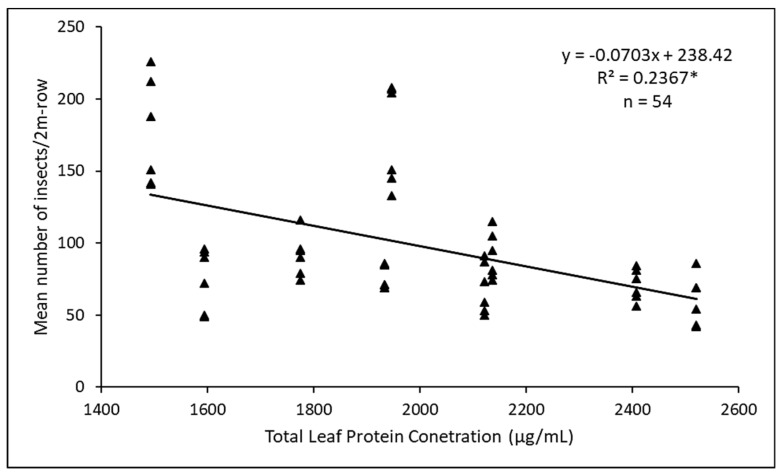
Regression analysis showing the relationship between total leaf protein content and *D. glabrata* population in 2018. * indicates significant regression coefficient at *p* < 0.05.

A Pearson correlation coefficient of −0.49 (n = 54, *p* < 0.0001) from the 2018 data shows a highly significant negative relationship between the *D. glabrata* population and total leaf protein content. In addition, the regression coefficient, r^2^, was significant (*p* < 0.05) with total leaf protein content accounting for about 24% of the observed variation in *D. glabrata* population (Figure 8).

### 3.5. Quantification of Total Leaf Polyphenol Content among Amaranth Varieties

**Figure 9:**
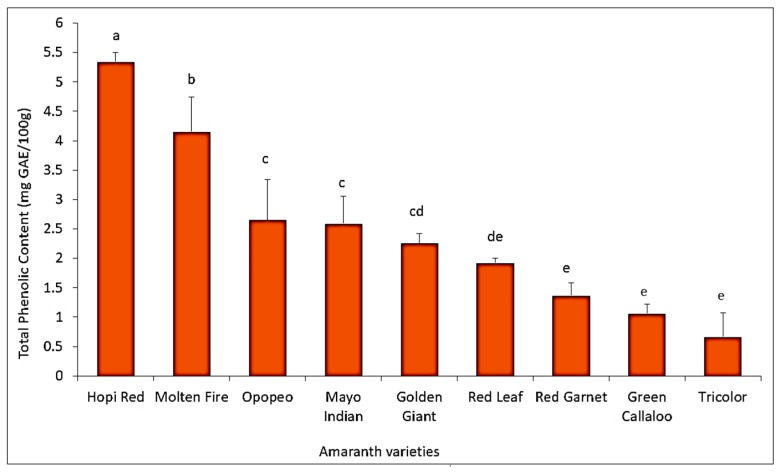
Total leaf polyphenol content (average ± SE) among amaranth varieties. Bars with the same letters are not significantly different using Fisher’s LSD (*p* < 0.05).

Significant differences were obtained in the total polyphenol content among the amaranth varieties. Two varieties, *Molten Fire* and *Hopi Red*, recorded the highest total leaf polyphenol content (>4 mg GAE/100g) which was significantly higher (F = 16.77, df = 8, 16; *p* < 0.0001) than in the susceptible varieties *Green Callaloo* (1.3 mg GAE/100g) and *Tricolor* (0.7 mg GAE/100 g) (Figure 9).

**Figure 10:**
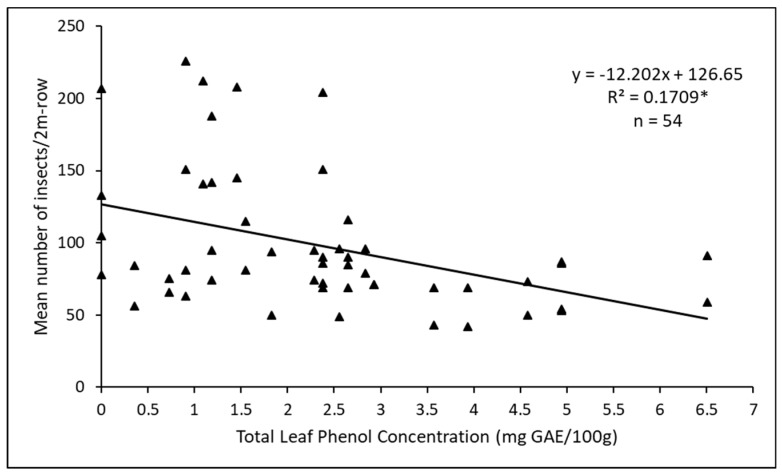
Regression line and equation showing the relationship between total leaf polyphenol content and *D. glabrata* population in 2018. * indicates significant regression coefficient at *p* < 0.05.

The Pearson correlation coefficient of −0.41 (n = 54, *p* < 0.0001) obtained from analyzing 2018 data shows a negative relationship between *D. glabrata* population and total leaf polyphenol content (Figure 10); the regression coefficient was significant (*p* < 0.05) with total leaf protein accounting for 17% of the observed variation in *D. glabrata* population.

### 3.6. Fresh Leaf Yield of Amaranth Varieties

Overall, mulching depressed the yield of greens in all varieties. The mean fresh leaf yield of amaranth differed significantly between plots with mulch, and those without mulch, and among subplots treatments (amaranth varieties) in both years. The mean fresh leaf yield recorded on plots without mulch in 2017 (F = 68.43, df = 1, 32; *p < 0.0001*) and in 2018 (F = 24.70, df = 1, 32; *p* < 0.0001) was 40% and 45%, higher compared to mulched plots. Regarding the amaranth varieties, *Mayo Indian (MI)* recorded the highest mean fresh leaf yield during both years (Table 4). The lowest yield values obtained were from *Tricolor (TR)* and *Green Callaloo (GC)*.

### 3.7. Dry Grain Yield of Amaranthus spp.

Six amaranth varieties namely, *Mayo Indian, Opopeo (OP), Red Garnet (RG), Hopi Red Dye (HR-D), Golden Giant (GG)*, and *Green Callaloo (GC)* were also assessed for grain production during both years of the study.

These results show that dry grain yield differed significantly between plots with mulch and those without mulch during both 2017 (F = 0.48, df = 1, 20; *p* = 0.0494) and 2018 (F = 12.46, df = 1, 20; *p* = 0.0021) (Table 5). Grain yield was 30% and 40% greater in plots without mulch compared to mulched plots during 2017 and 2018, respectively. *Mayo Indian (MI)* recorded the highest dry grain yield while the lowest was recorded in *Green Callaloo (GC)* in both years. Our results do not show significant interaction between mulching and amaranth grain yield in 2017 (F = 1.10, df = 5, 20; *p* < 0.3936) and in 2018 (*F* = 0.88, df = 5, 20; *p* < 0.5098). In 2018, two varieties, HR-D and TG had higher yields with mulching than without mulch.

### 3.8. Overall Ranking of Amaranth Varieties Based on Agronomic Performance

Amaranth varieties were ranked using pooled data from 2017 to 2018 based on four key agronomic parameters: (i) Mean damage score; (ii) key insect population; (iii) mean fresh leaf yield; and (iv) yield reduction due to mulching. Using the stated parameters, *Molten Fire*, *Red Garnet* and *Hopi Red* were ranked best performers with final ranks 1, 2 and 3, respectively. Using the same criteria, the three worst performing varieties and their respective ranks (in parentheses) were *Tricolor* (5), *Red Leaf* (6) and *Green Callaloo* (7) (Table 6). The varieties that were most susceptible to damage (CC and RL) were also the lowest ranked overall. The reverse was also true for the resistant (least damaged) varieties (MF and RG).

## 4. Discussion

### 4.1. Insect Diversity and Population on Amaranthus spp.

Among the insects reported on *Amaranthus* spp., those in the families, Agromyzidae (Diptera); Chrysomelidae and Curculionidae (Coleoptera); Blissidae, Cicadellidae, Miridae, Cercopidae, Membracidae, Acanaloniidae, Aphididae, Coreidae, Pentatomidae (Hemiptera); Acrididae (Orthoptera); Hespiridae, Crambidae (Lepidoptera) were found to cause significant damage [30,31,41]. Generally, amaranth pests can be classified into three major groups: defoliators (Coleoptera: Chrysomelidae), stem and root feeders (Coleoptera: Curculionidae; Lepidoptera: larval stage), and grain feeders (Hemiptera: Coreidae) [23,38,42]. In the present study, the most destructive insect pest observed on amaranth was the defoliator, *D. glabrata* (Coleoptera: Chrysomelidae). Both the larval and adult stage were observed to cause defoliation as has been reported by other studies in the United States [42,43]. The greatest damage was caused by adult *D. glabrata* which had also been reported defoliating large plots of amaranth in eastern Kentucky and New York by Stegmaier [42] and Garman [43], respectively. In addition, the studies of Lopez-Olguin et al. [44] and Vogt et al. [45] also reported *D. glabrata* as a major pest on plants in the Amaranthaceae family in North and South America, respectively. This is clearly the most serious pest of amaranth greens in this geographical zone. However, Kagali et al. [46] observed *Hylephila phyleus* and *Pieris rapae* as major pests of amaranth plants in Kenya. Lepidoptera were of much lower significance in our study. Amaranthus weevils, *Hypolixus truncatulus* and *Rhyssomatus aequalis*, found in the current study caused both primary damage and secondary invasion to stems of amaranth plants resulting in very limited plant lodging and death. Further, *Cletus* sp. observed by Aderolu et al. [38] as a major amaranth grain pest was not found to cause significant grain yield loss in our study. This could be due to early harvesting of the seed heads and indoor drying. Significantly, and rather curiously, is the apparent lack of any parasitoids among the samples. However, we found a few known families of generalist predators that prey on soil inhabiting and soft bodied insects; for example, Coccinelidae, Vespidae and Carabidae. Collectively they seem to represent a rather small functional group in the investigated phytobiome. A possible explanation may be the fact that *D. glabrata* larvae were not an important factor in the observed defoliation and thus were not given enough priority in our study; for example, no pitfall trap or Berlese funnel sampling was carried out. That would probably have provided more information on the ground surface predator-prey dynamics. Future studies will need to focus more on these aspects of *D. glabrata* biology in mulched and bare soil environments.

### 4.2. The Role of Leaf Mulch on D. glabrata Population

Insects vary in their response to different mulches and the sampling methods used [7,23]. Several studies have shown that organic mulches influence the populations levels and diversity of insects [29,47,48]. The study by Gill and Goyal [29] reported more diversified and higher insect populations on *Antirrhinum majus* L. mulch treated plots, compared to those with no mulch. Mochiah et al. [48] also reported similar results in their *Capsicum annuum* l. study. The results from the current study are in concurrence with these studies in that higher insect diversity (*H′* = 2.3) was recorded on the amaranth plots with mulch (2.0 in plots without mulch). From these results we can surmise that mulch alters the microenvironment and favors increased growth and development of the arthropods in question and may thus have accounted for the above scenario [29]. Some workers have conjectured that the dark color of the mulch may have attracted these insects [49]; presumably, other factors such as humidity profile and odor may have also been important in this regard. In our study, mulched plots had higher moisture content than where there was no mulch and higher *D. glabrata* numbers were observed on mulched plants. This may suggest the lack of repellence in the specific leaf compost mulch used, even as a suitable microhabitat was created [47]. Furthermore, the C:N ratio (of 40:1) of the leaf mulch that was used compared to other materials such as wood (with a C:N ratio of 400:1) made nitrogen more readily available thus accounting for higher plant vegetative growth, especially leaf and tissue biomass [29,50]. The abundance of leaf/tissue biomass significantly supported the growth, development, and reproduction of *D. glabrata* hence the high populations observed. Prevention of desiccation and the promotion of metabolic activities provided by the moisture in the leaf mulch may also have contributed to the high beetle populations [28]. However, contrary to our findings, some workers have reported a decline in insect populations associated with the use of organic mulch. Johnson et al. [51] and Stoner et al. [52] observed a lower population of the Colorado potato beetle (*Leptinotarsa decemlineata* Say) when straw mulch was used. Gill et al. [6] also observed a significant decrease in the population of the lesser cornstalk borer (*Elasmopalpus lignosellus* (Zeller) on plots with straw mulch. The relatively high C:N ratio of the straw mulch (80:1) in comparison to the leaf mulch (40:1), makes nitrogen immobile for plant vegetative growth. As earlier indicated, this translates in less availability of plant tissue for insect growth and development [6]. In addition, the density of the straw applied may serve as a surface barrier preventing larval migration after eclosion below the soil surface, but this may have differential importance among insect species. It is also possible that moisture retained by straw mulch may lead to the production of compounds that may be toxic to the insects in question; this would result in lower populations compared to a cropped area without straw. In many of the studies cited earlier [11,12,13,14,15,16,17], mulching reduced pest numbers generally as a result of enhanced natural enemy activity. If one were to draw a parallel with this study, one would have to conjecture that the beneficial fauna in the case of amaranth and *D. glabrata* was simply at a very low ebb to exert significant any impact on the adult beetle population. Several reasons could be adduced for this, including interference with mobility and other activity of natural enemies as proposed by Thomson and Hoffmann [13] among others.

### 4.3. Herbivory on Cultivated Amaranthus spp.

The differences among the amaranths evaluated points clearly to the fact that some varieties were more suitable for feeding and development (highly susceptible to damage), whereas others were much less suitable (resistant to damage). This may be explained mainly by the observed biochemical and possibly morphological differences. Among the amaranth varieties, *Green Callaloo*, *Tricolor* and *Red Leaf* cultivated mainly for their leaves recorded the highest *D. glabrata* population and leaf damage. Their green leaf coloration (which signals high chlorophyll and sugar content) would render them more suitable for feeding thus favoring increased consumption by *D. glabrata* [53]. Varieties, such as *Molten Fire*, *Hopi Red Dye*, *Red Garnet*, *Opopeo* and *Mayo Indian* with reddish leaf coloration signifying the presence of anthocyanins [24,54] were resistant to feeding and received much less damage. Anthocyanins have been reported to influence plant defense against herbivores either through direct toxicity from ingestion, or possibly as a warning or by cryptic coloration [24]. Amaranth plants, however, do not contain anthocyanins, but rather betalains, a class of yellow and red pigments that are found in the Caryophyllales, and are responsible for the reddish coloration of the plant parts and exhibit similar effects [24]. Some insects, possibly including *D. glabrata*, tend to avoid plants with such coloration; the low populations and damage recorded on these varieties may be in part be explained by this trait. Furthermore, from a resource availability perspective, the tiny and small number of leaves observed in *Molten Fire* variety compared to the relatively broad and numerous leaves on *Green Callaloo* variety may also have accounted for the former receiving a minimum of damage [31]. Sclerophylly, the presence of toughened and hardened leaves among the grain producing varieties may also have played a role in the low *D. glabrata* incidence and low damage [53], but we did not determine this conclusively. Also, none of the varieties had trichomes.

Research on *Amaranthus* leaf flavonoid content carried out by Niveyro et al. [24] and Kalinova and Dadakova [55] found rutin, nicotiflorin, isoquercetin, gallic acids and betalains; amaranthine, iso-amaranthine, betanin, glycine, betaine, trigonelline in various concentrations. Even though these compounds do not affect the normal growth and development of the plants, their individual concentrations and interactions with other compounds can, either increase or reduce the palatability of plants. This confers either, resistance or susceptibility to insects [53] as can be deduced for example on *Green Callaloo* and *Red Leaf* (susceptible), and for *Molten Fire, Hopi Red, Red Garnet* (resistant). The variation in leaf damage observed among the amaranth varieties would support this, as may be the presence and/or balance of the other compounds, such as betalains that were identified in this, or other, studies [53,54,55,56].

Many polyphenols and plant proteins are known to render plants resistant or susceptible to insects depending on their concentrations in plants [24,53]. Our results seem to confirm this and show a significant negative correlation between total polyphenols and leaf proteins with *D. glabrata* population and herbivory (Figure 8). For example, *Green Callaloo* (susceptible variety) and *Molten Fire* (resistant variety) recorded the lowest, and highest total leaf protein concentration, respectively (Figure 9). This generally corroborates the work of Huerta-Ocampo et al. [57] who concluded that high protein content present in amaranth plants may contribute to their resistance to abiotic stressors and vice versa. Apart from the concentration, the type of polyphenol or protein is also important in this regard [53,56]. Variation in the quality of plant nutrients influences herbivory as insects usually consume more to compensate for a lower quality diet [53]. We believe this may be the case with the susceptible *Green Callaloo*, with the lowest total leaf protein content, but a high level of herbivory.

### 4.4. Effects of Organic Mulch on Fresh Leaf and Dry Grain Yield of Amaranthus spp.

Mulching has the potential to influence crop yield, either directly or indirectly [6,11,13,14,16,17]. The type of mulch used is important and may have played a significant role in our findings. According to Rahman et al. [58] mulching with plant residue increases the profitability of many crops by improving the soil organic matter. Synthetic inorganic mulches, such as plastics, fabrics and reflective aluminum mulch, on the other hand, do not significantly increase yield, but are thought to be better than having no mulch at all, due to their ability to significantly reduce weeds and some insect pests, as well as conserve moisture [58]. This conclusion cannot however be made in the case of *D. glabrata* in which leaf compost mulch increased the pest population and crop damage and also drastically reduced green leaf yield. We did not evaluate plastic or other synthetic mulches. The leaf compost mulch used in the present study increased soil organic matter, provided optimum soil moisture and temperature necessary for vegetative plant growth, it clearly created an ideal microclimate for growth and development of the *D. glabrata*. The observed differences in yield among the amaranth varieties may also be attributed to differences in their genetic traits, such as the biochemical makeup, morphological characteristics and other related variables. It is instructive that the highest ranked varieties in Table 6 were also the most resistant and had the least yield reduction. It would be interesting to know how these varieties rank in consumer acceptability.

## 5. Conclusions

All the *Amaranthus* spp. used in this study have great potential for production and income generation for growers in NC; they are all edible and increasingly popular. Several insect pests were observed on *the amaranths*; however, *D. glabrata* was the most destructive causing significant damage and yield loss. The leaf mulch used in the study increased the population of *D. glabrata* that resulted in higher leaf damage. Compounds that were identified including plant proteins and polyphenols, as well as other possible compounds may account, at least in part, for the differences observed in damage among the different amaranths.

Based on our findings, we would caution that despite the popularity of mulches in organic culture to enhance natural enemy activity and weed management, as well as other ecological services [11,13,14,16], growers need to ensure that their decision, whether to use or not to use mulch, is based on sufficient knowledge regarding its interaction with specific crops and its impact on key pests in their respective systems. Mulches do not all work the same way in all systems. As shown very clearly in this study, leaf compost mulch will increase losses in amaranth production, especially for varieties that are highly susceptible to leaf damage by flea beetles such as *D. glabrata*. We cannot guarantee that using another type of mulch will have the same results as those observed in this case; therefore, the decision to use or not to use mulch must be made on a case-by-case basis. Other mulches, such as fabrics and plastics as well as those of plant origin (e.g., straw) may be appropriate but should be adequately evaluated before they are used.

## Figures and Tables

**Table 1:** Morphological traits of amaranth varieties used in the study.

Variety^1^	Species (Type ^2^)		Plant Architectural Traits						
	*Amaranthus*	Origin	Growth Habit ^3^	Maturity ^4^	Leaf Color ^5^	Leaf Shape ^6^	Leaf Pubescence ^7^	Branch Type ^8^	Inflorescence Type ^9^ Color ^10^
TR	*tricolor* (V)	Asia	1	30–50	2	3	1	1	n/a n/a
MF	*tricolor* (V)	Asia	1	30–50	3	3	1	1	n/a n/a
GC	*viridis* (V/G)	China	1	90–120	1	2	1	3	1 1
HR-D	*hypochondiacus* *(V/G)*	USA	2	65–75	2	1	1	2	1 2
GG	*cruentus* (G/V)	USA	1	98–110	1	2	1	2	2 1
OP	*cruentus* (G/V)	Mexico	1	62–75	3	3	1	4	2 3
RG	*cruentus* (G/V)	Mexico	1	75–110	3	3	1	4	2 3
RL	*cruentus* (G/V)	Asia	1	50	2	4	1	4	3 1
MI	*cruentus* (G/V)	Mexico	1	90	2	2	1	3	2 3

^1^ TR = Tricolor, MF = Molten Fire, GC = Green Callaloo, HR-D = Hopi Red Dye, GG = Golden Giant, OP = Opopeo, RG = Red Garnet, RL = Red Leaf, MI = Mayo Indian; ^2^ V = Vegetable, G = Grain; ^3^ 1 = erect, 2 = prostrate; ^4^ = Days to maturity; ^5^ 1 = green, 2 = green-variegated red or red-variegated green, 3 = red purple; ^6^ 1 = elliptic, 2 = ovate, 3 = lanceolate, 4 = cordate, 5 = oblong; ^7^ Leaf pubescence and plant habit were assessed on insect-free 90-day old plants, 1 = glabrous; ^8^ 1=unbranched, 2=few branches all near the base of stem, 3= many branches all near the base of the stem, 4= branches all along the stem; ^9^ 1 = intermediate, 2 = Dense, 3 = Lax.; ^10^ 1 = green, 2 = red-purple, 3 = red.

**Table 2:** List of insects on amaranth plots June 2017 to August 2018.

Order	Family	Scientific Name	Common Name ^1^	Number of Insects 2017		Number of Insects 2018	
				Mulch	No Mulch	Mulch	No Mulch
Hemiptera	Blissidae	*Blissus leucopterus*	Chinch Bug	19	9	19	12
	Cicadellidae	*Graminella nigrifrons*	Leafhopper	500	300	400	157
		*Graphocephala* sp.		50	30	50	30
	Miridae	*Lygus* sp	Tarnish Plant Bug	115	108	100	98
	Cercopidae	*Prosapia bicincta*	Spittle Bug	30	4	21	18
	Membracidae	*Ceresa sp*.	Treehopper	62	18	50	20
	Acanaloniidae	*Acanalonia servillei*	Planthopper	39	7	30	27
	Aphididae	*Aphis nerii*	Aphid	660	278	472	192
	Coreoidea	*Cletus* spp.	Horned Coreid Bug	11	6	20	27
	Pentatomidae	*Cosmopepla lintneriana*	Twice-stabbed Stink Bug	23	3	34	8
Coleoptera	Coccinelidae	*Coleomegilla maculate* **^+^**	Spotted Lady Beetle	88	109	60	85
		*Harmonia axyridis* **^+^**	Multicolored Asian Lady Beetle	25	28	30	20
	Carabidae	*Lebia analis **^+^***	Ground Beetle	44	29	50	20
	Chrysomelidae	*Disonycha glabrata*	Pigweed Flea Beetle	980	558	850	508
		*Deloyata guttata*	Tortoise Beetle	19	17	22	22
		*Diabrotica undercimpunctata*	Spotted Cucumber Beetle	20	31	20	31
		*Acalymma vittatum*	Stripped Cucumber Beetle	20	24	2	24
	Curculinodae	*Rhyssomatus aequalis*	Amaranthus Weevil	14	20	12	25
		*Hypolixus truncatulus*		10	18	8	15
	Myleridae	*Collops quadrimaculatus*	Soft-winged Flower Beetle	11	10	20	20
	Elateridae	*Megapenthes insignis*	Click Beetle	10	0	19	30
Diptera	Agromyzidae	*Agromyza* spp.	Leaf miner	100	0	98	80
	Do lichopodidae	*Condylostylus* sp.**^+^**	Long Legged Fly	102	120	82	200
	Sarcophagidae	*Ravinia sp. º*	Flesh Fly	30	26	52	0
Lepidoptera	Hespiridae	*Hylephila phyleus*	Skipper	11	12	11	21
	Pieridae	*Pieris rapae*	Small Cabbage Butterfly	3	5	3	15
Hymenoptera	Vespidae	*Ancistrocerus sp.º*	Yellow Jacket Wasp	12	10	12	11
		*Euodynerus sp*.	Brown Paper Wasp	10	9	10	30
		*Pachodynerus erynnis*	Hornet	5	1	5	0
	Halictidae	*Augochloropsis metallica ^−^*	Sweat Bee	10	0	12	24
Orthoptera	Acrididae	*Chortophaga viridifasciata*	Green Grasshopper	8	6	20	0
			**Total**	3038.0 ± 42.7	1796.0 ± 22.3	2594.0 ± 35.5	1649.0 ± 19.7
			**Shannon Weaver Diversity Index (*H′*)**	2.3	2.1	2.3	2.0
			**Index of Evenness (*E*)**	0.23	0.19	0.67	0.59

^1^ Committee on the common names of insects. Entomological Society of America; **^+^**/ **º / ^−^** Beneficial insects: **^+^** Predator; **º** Parasitoid, **^−^** Pollinator.

**Table 3:** Damage scores (Mean ± SE) of amaranth varieties in mulch and no mulch treatments.

Amaranth Variety	Damage Scores ^1^ (2017)		Damage Scores ^1^ (2018)	
	Mulch	No Mulch	Mulch	No Mulch
Tricolor (TR)	1.4 ± 0.09 ^c^A	1.3 ± 0.14 ^c^B	1.4 ± 0.08 ^cb^A	1.3 ± 0.15 ^cb^B
Green Callaloo (GC)	3.7 ± 0.24 ^a^A	2.2 ± 0.12 ^a^B	3.8 ± 0.12 ^a^A	2.4 ± 0.13 ^a^B
Hopi Red Dye (HR-D)	1.3 ± 0.07 ^b^A	1.3 ± 0.09 ^b^B	1.2 ± 0.05 ^cb^A	1.1 ± 0.07 ^cb^B
Golden Giant (GG)	1.6 ± 0.19 ^b^A	1.6 ± 0.19 ^b^B	1.5 ± 0.07 ^cb^A	1.2 ± 0.10 ^cb^B
Opopeo (OP)	1.6 ± 0.15 ^b^A	1.0 ± 0.03 ^b^B	1.6 ± 0.03 ^b^A	1.5 ± 0.03 ^b^B
Molten Fire (MF)	1.0 ± 0.03 ^c^A	1.2 ± 0.03 ^c^B	1.2 ± 0.03 ^c^A	1.0 ± 0.03 ^c^B
Red Garnet (RG)	1.5 ± 0.03 ^b^A	1.5 ± 0.02 ^b^B	1.8 ± 0.03 ^cb^A	1.2 ± 0.03 ^cb^B
Red Leaf (RL)	3.6 ± 0.25 ^b^A	2.3 ± 0.06 ^b^B	3.8 ± 0.20 ^a^A	1.5 ± 0.03 ^a^B
Mayo Indian (MI)	1.5 ± 0.04 ^a^A	2.0 ± 0.06 ^a^B	1.5 ± 0.17 ^b^A	1.5 ± 0.09 ^b^B

Means followed by the same upper-case letters (within rows) or lower-case letter (within columns) are not significantly different at *p* < 0.05 using Fisher’s LSD (*p* < 0.05) ^1^ Damage Scores recorded on a visual scale of: 1 = 0–20%; 2 ≧ 20–40%; 3 ≧ 40–60%; 4 ≧ 60–80%; 5 ≧ 80–100% leaf damage.

**Table 4:** Marketable fresh leaf yield (Mean ± SE) of amaranth varieties in mulch and no mulch treatments.

Amaranth Variety ^1^	Mean Fresh Leaf Yield ^1^ (Kg/ha) (2017)		Change in Yield Due to Mulching	Mean Fresh Leaf Yield ^1^ (Kg/ha) (2018)		Change in Yield Due to Mulching
	Mulch	No Mulch	(%)	Mulch	No Mulch	(%)
TR	9298.6 ± 901.9 ^e^A	10911.4 ± 950.6 ^e^B	−14.8 ± 3.9	6388.9 ± 734.9 ^a^A	7496.9 ± 819.0 ^b^B	−14.8 ± 2.9
GC	9651.4 ± 1277.2 ^de^A	13103.8 ± 908.6 ^de^B	−26.3 ±15.8	4333.3 ± 693.9 ^a^A	9215.6 ± 1033.5 ^b^B	−53.0 ± 3.7
HR-D	13985.8 ± 1200.9 ^cd^A	17790.9 ± 1640.3 ^cd^B	−21.4 ±21.0	9666.7 ± 787.6 ^a^A	11012.6 ± 669.5 ^b^B	−12.2 ± 1.9
GG	14892.9 ± 226.8 ^b^A	21369.2 ± 643.5 ^b^B	−30.3 ± 3.0	12166.7 ± 1205.7 ^a^A	14237.7 ± 2336.0 ^b^B	−14.5 ± 6.1
OP	19050.9 ± 1209.6 ^a^A	23612.0 ± 1034.1 ^a^B	−19.3 ± 1.7	12333.3 ± 838.9 ^a^A	13960.6 ± 1340.7 ^b^B	−11.7 ± 2.8
MF	9323.8 ± 504.0 ^cd^A	14666.1 ± 615.7 ^cd^B	−36.4 ± 4.3	8277.8 ± 1010.7 ^a^A	9240.7 ± 990.8 ^b^B	−10.4 ± 1.6
RG	15422.1 ± 1229.1 ^b^A	20865.2 ± 1442.3 ^b^B	−26.1 ± 1.1	12231.3 ± 846.8 ^a^A	13542.2 ± 1458.7 ^b^B	−9.7 ± 3.7
RL	12070.6 ± 995.6 ^c^A	16455.3 ± 1019.3 ^c^B	−26.6 ±10.3	5000.0 ± 1734.7 ^a^A	9400.2 ± 1918.3 ^b^B	−46.8 ± 7.5
MI	20361.2 ± 613.1 ^a^A	26610.7 ± 1930.9 ^a^B	−23.4 ± 6.1	1413.70 ± 891.3 ^a^A	1569.3 ± 857.9 ^b^B	−9.9 ± 0.8

Means followed by the same upper-case letters (within rows) or lower-case letter (within columns) are not significantly different (Fisher’s LSD; *p* < 0.05); ^1^ Mean fresh leaf yield values extrapolated from 8 plants per plot. TR = Tricolor, MF = Molten Fire, GC = Green Callaloo, HR-D = Hopi Red Dye, GG = Golden Giant, OP = Opopeo, RG = Red Garnet, RL = Red Leaf, MI = Mayo Indian.

**Table 5:** Marketable dry grain yield (Mean ± SE) of amaranth varieties in mulch and no mulch treatments.

Amaranth Variety ^1^	Mean Dry Grain Yield ^1^ (Kg/ha) (2017)		Change in Yield Due to Mulching	Mean Dry Grain Yield ^1^ (Kg/ha) (2018)		Change in Yield Due to Mulching
	Mulch	No Mulch	(%)	Mulch	No Mulch	(%)
GC	222.2 ± 55.6 ^a^A	444.4 ± 111.1 ^a^B	−50.0 ± 0.0	1000.0 ± 384.9 ^c^A	1333.3 ± 192.5 ^b^B	−25.0 ± 0.0
HR-D	333.3 ± 96.2 ^ab^A	555.6 ± 147.0 ^ab^B	−40.0 ± 0.0	1277.8 ± 618.6 ^c^A	833.3 ± 419.0 ^c^B	53.3± 0.0
GG	1833.3 ± 96.2 ^ab^A	1944.4 ± 200.3 ^ab^B	−5.7 ± 0.1	1777.8 ± 111.1 ^b^A	2055.6 ± 242.2 ^b^B	−13.5 ± 0.0
OP	1888.9 ± 147.0 ^ab^A	1944.40 ± 147.0 ^ab^B	−2.9 ± 0.0	1722.2 ± 55.6B ^b^A	1722.2 ± 55.6 ^b^B	0.0 ± 0.0
RG	1388.9 ± 277.8 ^ab^A	1777.8 ± 111.1 ^ab^B	−21.9 ± 0.3	2500.0 ± 481.1 ^b^A	1611.1 ± 200.3 ^b^B	55.2± 0.0
MI	2555.6 ± 337.9 ^b^A	3388.9 ± 55.6 ^b^B	−24.6 ± 0.4	3111.1 ± 309.3 ^a^A	3611.1 ± 419.4 ^a^B	−13.9 ± 0.0

Means followed by the upper-case letters within rows or lower-case letter within columns are not significantly different at *p* < 0.05; Fisher’s LSD (*p* ≤ 0.05); ^1^ Mean dry grain yield values extrapolated from 8 plants per plot. GC = Green Callaloo, HR-D = Hopi Red Dye, GG = Golden Giant, OP = Opopeo, RG = Red Garnet, MI = Mayo Indian.

**Table 6:** Ranking of Amaranth Varieties based on Agronomic Performance.

Amaranth Varieties	Damage Score ^1^		Insect Population ^1^		Yield ^1^ (Kg/ha)		Yield Reduction Due to Mulching ^1^ %	Total Sum of Rank	Final Rank
	M ^2^	NM ^3^	M ^2^	NM ^3^	M ^2^	NM ^3^			
Molten fire (MF)	1	1	1	2	7	7	2	21	1
Red Garnet (RG)	2	3	6	7	3	4	2	27	2
Hopi Red Dye (HR-D)	2	3	3	2	6	5	7	28	3
Opopeo (OP)	5	5	3	7	2	3	6	31	4
Mayo Indian (MI)	5	5	7	7	2	1	4	31	4
Golden Giant (GG)	5	4	7	6	2	3	4	31	4
Tricolor (TR)	3	3	3	4	8	9	6	32	5
Red Leaf (RL)	5	5	7	6	7	6	6	42	6
Green Callaloo (GC)	7	6	9	9	8	7	9	55	7

Lower values more desirable (i.e., better performance) than higher values. ^1^ Mean values are for 2017 and 2018 results; ^2^ M = Mulch treatment; ^3^ NM = No mulch treatment.
